# Anticancer Properties of Chrysin on Colon Cancer Cells, *In vitro* and *In vivo* with Modulation of Caspase-3, -9, Bax and Sall4

**DOI:** 10.15171/ijb.1374

**Published:** 2016-09

**Authors:** Maliheh Bahadori, Javad Baharara, Elaheh Amini

**Affiliations:** ^1^Department of Biology, Damghan Branch, Islamic Azad University, Damghan, Iran; ^2^Department of Biology, Research Center For Animal Development Applied Biology, Mashhad Branch, Islamic Azad University, Mashhad, Iran; ^3^Department of Animal Biology, Faculty of Biological Sciences, Kharazmi University, Tehran, Iran

**Keywords:** Apoptosis, Chrysin, Colon cancer, *In vivo*, Sall4

## Abstract

**Background:**

The SALL4/Sall4 is constitutively expressed in human and mice. SALL4 mRNA could be used as a marker for the diagnosis of different types of cancers. On the other hand, chrysin has diverse biological properties.

**Objectives:**

In the present study, the effect of the chrysin was investigated on the CT26 colon cancer *in vitro* and *in vivo*. Furthermore, the expression levels of the stem cell markers; sall4 and Bax was analyzed, as well.

**Materials and Methods:**

The cytotoxic effects and the type of cell death induced by chrysin were evaluated using a number of biological assays. The apoptotic pathway was examined by caspase-3and caspase-9 assay. The *in vivo* antitumor efficacy of chrysin on transplanted CT26 tumor cells in BALB/c mice was investigated. In addition, mRNA expression of sall4, Bax was analyzed with RT-PCR.

**Results:**

MTT assay and morphological characteristics showed that chrysin exerted a cytotoxic effect on CT26 cells in a dose dependent manner with IC_50_= 80 μg.mL^-1^. The biological assays have indicated that chrysin administrated cytotoxicity on colon cancer cells through recruitment of the apoptosis. Caspase-3 and caspase-9 colorimetric assays, in addition to Bax expression analysis, have indicated the involvement of intrinsic apoptotic pathway in the cytotoxic effect of the chrysin. The *in vivo* assay revealed a remarkable reduction of the colon tumor volume in treated mice (8, 10 mg.kg ^-1^) as compared to the untreated mice. RT-PCR elucidated that chrysin attenuated tumor volume through down regulation of the sall4 and up-regulation of the Bax.

**Conclusions:**

It was demonstrated that chrysin accomplishes anti-cancer effect on colon cancer cells via induction of the apoptosis and attenuation of the sall4 the expression. These findings introduce chrysin as an efficient apoptosis based therapeutic agent against colon cancer.

## 1. Background

Colon cancer is a world-wide health problem and‏ the second most lethal cause of cancer-related death‏ both males and females in the world. It afflicts more‏ than 135,000 patients per year in the world with a incidence‏ rate of 9.4% and kills more than 55,000 patients‏ (1). Colorectal cancer ranks the second among the‏ most tumor incidence death (2). The incidence of the‏ colorectal cancer is 8.5% of all cancers and may be‏ related to the lifestyle, obesity, drinking, smoking, as‏ well as other factors (3).‏

The traditional treatment of the colon cancer‏ includes assisting (application or administration)‏ chemotherapeutic agents and surgery, depending on‏ the location of the metastatic growth, however, the‏ current chemotherapeutic agents induce serious side‏ effects and high toxicity, which also increase the risk‏ of death (4). Chemotherapy is one of the most important‏ treatments for cancer patients. However, the efficacy‏ of the chemotherapeutic drugs is still limited in‏ most solid tumors (5).

There are more than one thousand different drugs‏ that were found to possess significant anticancer properties‏ (6). However, there is still continues demand for‏ development of the new anticancer drugs by scientific‏ exploration among a vast variety of synthetic, biological,and natural products (7).

Natural products have been used as an important‏ source of anti-cancer agent, which is estimated to‏ become the major alternative for reducing the cancer‏ death in the 21st century (6).

Pro-apoptotic potential of the natural substances‏ has made them candidate against cancer (8). Plants‏ represent wide variety of phytochemicals with pharmaceutical‏ potential such as poly phenolic, terpenoids,‏ taxol, podophyllum peltatam, and flavonoid compounds.‏ Flavonoids are a class of compound composed‏ of a wide range of plant pigments that are universally‏ present in the fruits and vegetables derived foods. To‏ date more than 4,000 types of biologically active‏ flavonoids have been identified (9). Chrysin is composed‏ of a large class of polyphenolic compounds‏ present in the diet and many herbal products, which‏ have long been associated with a variety of important‏ biochemical and pharmacological activities in the cancer‏ prevention and health promotion (9,10).

A number of studies have shown that chrysin has‏ multiple biological activities, such as anti-inflammation,‏ anti-oxidation and anticancer effects (10). In‏ addition, chrysin has been reported to induce apoptosis‏ in a panel of cancer cell lines, including Hela cervical‏ cancer cells, U937, HL-60, and L1210 leukemia cells.‏ This plant flavonoid was also able to inhibit tumor‏ angiogenesis *in vivo*, which is a key step in cancer cell‏ metastasis (11). It has been reported that chrysin possess‏ cytotoxic effect on human colon cancer *in vitro* (9).

The SALL gene family has been found to be‏ involved in tumorigenesis. This gene family is among‏ the most important transcriptional regulators of the‏ stem cells. SALL4 is the most well studied SALL gene‏ associated with the cancer s. Recent studies have‏ shown that SALL4 is an excellent target for assessment‏ of treatment with regard to several types of cancers‏ including, liver, ovarian primitive germ cell, and‏ testicular germ cell tumors (12). The association‏ between SALL4 and OCT4, NANOG, and SOX2 suggests‏ that SALL4 might be a candidate marker for the‏ metastatic germ cell tumors (13). SALL4 was highly‏ expressed in several solid tumors such as breast cancer,‏ germ cell tumor, hepato and gastric cancers, as‏ well as colon carcinoma (14). The role of SALL4 was‏ the focus on carcinogenesis, prevention, treatment of‏ tumor cell lines, and tumor tissues (15).

Our aim was to investigate the anti-cancer effect of‏ chrysin on CT26 colon cancer cells both *in vitro* and *in vivo*. Furthermore, we were interested in achieving‏ more insights on the role of chrysin through evaluation‏ of the type of cell death it induces in CT26 cells; in‏ addition to examining the expression of sall4 in colon‏ cancer cells under treatment with chrysin, as well.

## 2. Objectives

In order to have a new insight into the mechanistic‏ cytotoxicity of chrysin, the present study was designed‏ to evaluate whether chrysin was cytotoxic against‏ CT26 colon cancer cells via involvement sall4 and caspase‏ dependent pathway.

## 3. Materials and Methods

### 3.1. Cell Culture

CT26 cells were procured from Pasteur Institute‏ (Tehran, Iran). The cells were cultured in RPMI-1640‏ medium, supplemented with 10% (v/v) Fetal Bovine‏ Serum (FBS, Gibco, Scotland), supplemented with the‏ 1% antibiotic and incubated at 37ºC in a humidified‏ atmosphere of 5% CO_2_.

### 3.2. In vitro Assays

#### 3.2.1. Cytotoxicity Assay

CT26 cells were seeded in a 96- well plate‏ overnight and treated with various concentrations of‏ chrysin (10, 20, 40, 80, 100, and 200 μg.mL^-1^) for 24‏ h and 48 h, MTT [3-(4,5-Dimethylthiazol-2-yl)-2,5-‏ Diphenyltetrazolium Bromide] (5 mg.mL^-1^ in PBS)‏ was then added to the cells and incubated in the dark‏ for 4 h. Finally, DMSO (dimethyl sulfoxide) was‏ added and optical density was measured at 560 nm‏ with an microplate spectrophotometer (epoch from Bio‏ Tack, USA) (16).

### 3.3. Morphological Assessments

CT26 cells were treated with various concentrations‏ of chrysin (10, 20, 40, 80, 100, and 200 ‏ μg.mL^-1^)‏ for 48 h and morphological alteration were observed‏ under an inverted microscope (Bio Photonic, Brazil)‏ and photographs were taken.‏

### 3.4. Apoptosis Evaluation Using PI (Propodium Iodide) Staining

CT26 cells were treated with inhibitory concentrations‏ of chrysin (40 and 80 μg.mL^-1^, respectively) for‏ 48 h. Cells were harvested and washed with PBS.‏ Afterwards, centrifugation was carried out, and the cell‏ pellet was incubated in 500 μL of a hypotonic buffer‏ (500 μg.mL^-1^ PI in 0.1% sodium citrate and 0.1%‏ Triton X-100) overnight at 4°C and flow cytometry‏ was performed using a BDFACS Calibur system‏ (Becton Dickinson, USA) (17).‏

### 3.5. Apoptosis Analysis with Annexin V-FITC and Propodium Iodide Method

For quantitative evaluation of the chrysin induced‏ apoptosis, Annexin/Propodium Iodide assay (Abcam,‏ UK) was performed according to the manufacture‏ instruction. Briefly, 48 h after treatment, the CT26‏ cells were trypsinized and resuspended in 500 μL of‏ the 1X binding buffer. Thereafter, 5 μL of Annexin-VFITC‏ and 5 μL of Propodium Iodide were added and‏ incubated at room temperature for 5 min, subsequently‏ analysis was conducted using FACSCalibur (Becton‏ Dickinson) flow cytometry (18).

### 3.6. Apoptosis Assessment by DAPI Staining

The cells (1.5×105) were seeded in the 6-well plate‏ and treated with different concentrations of chrysin for‏ 2 days. The cells were washed twice with PBS, and‏ then DAPI [2,2’- diphenyL^-1^-picrylhydrazyl] was‏ added and incubated for 10 min in the dark. Finally the‏ cells were washed twice with PBS, resuspended in 1‏ mL methanol, and the nuclear morphology was‏ observed under a fluorescence microscopy (18).

### 3.7. Caspase-3 and Caspase-9 Asctivity

In order to detect the induced apoptotic pathway, caspase-9 or caspase-3 colorimetric assay (Abcam-UK) was‏ conducted as per manufacturer’s instruction. CT26 cells‏ (2×06) were treated with inhibitory concentrations of‏ chrysin for 48 h. Then cells were harvested and mixed‏ with 50 μL lysis buffer. Subsequently, 50 μL of 2X reaction‏ buffer was added to each cytosolic extract. Finally,‏ 5 μL of DEVD-p-NA (caspase-3 substrate) or LEHDpNA‏ (caspase-9 substrate) were added and the‏ absorbance of samples was read at 405 nm (19).

### 3.8. In vitro Assays

#### 3.8.1. Chrysin Preparation

Chrysin was purchased from Sigma (Cat no. C‏ 80105). To prepare different concentrations of the‏ chrysin (0.5, 1, 2, 4, 8, and 10 mg.kg^-1^), chrysin was‏ dissolved in 100 μL DMSO (Merck, Germany) and‏ was diluted with serum before experiments.

#### 3.8.2. Animals

Male BALB/c mice (25±5 g, 6 weeks of age) were purchased‏ from Pasteur Institute of Iran, and were fed with‏ chow diet (Tous, Khorasan). The mice were maintained in‏ standard condition at 25±2ºC, 12 h light/dark cycles with a‏ relative humidity of 55±5% and a 12 h time cycle.

### 3.9. In vivo Antitumor Assessment

‏ To create an allograft colon carcinoma model, CT-‏ 26 mouse colon cancer cells (1.5×10^6^ cells in 0.2 mL‏ PBS) were injected subcutaneously into the right flank‏ of BALB/c mice (n=5). After subcutaneous tumor formation‏ (approximately 7 days) the animals were randomly‏ designated into 5 groups of 5 mice.‏

The mice were divided into group I (control, administrated‏ PBS orally duration treatment period) and treatment‏ groups received chrysin dissolved in serum including group‏ II (0.5 mg.kg^-1^ chrysin), group III (1 mg.kg^-1^ chrysin),‏ group IV (2 mg.kg^-1^ chrysin extract), group V (4‏ mg.kg^-1^ chrysin extract), group VI (8 mg.kg^-1^ chrysin),‏ and group VII (10 mg.kg^-1^ chrysin) via oral administration‏ once daily for 2 weeks. The mice were sacrificed at‏ the end of 2 week and the tumors were removed, then‏ fresh tumors immediately were kept in an RNAlater‏ solution (Qiagen, Hilden, Germany) at -20ºC until‏ extraction.

### 3.10. Total RNA Extraction, cDNA Synthesis, and Quantitative RT-PCR

The untreated and treated tumor tissues were snap‏ frozen in the liquid nitrogen, ground mechanically to a‏ fine powder, and were placed in the new cryogenic‏ tubes. Then, RNA was extracted from colon tissue‏ samples using the RNeasy kit according to manufacturer’s‏ instruction (Denazist, IRAN), cDNA was synthesized‏ using cDNA synthesis Kit following to the‏ application of total extracted RNA plus oligo dT‏ (Oligo(dT))_12-18_ Primer is suitable for use in firststrand‏ cDNA synthesis with reverse transcriptase. The‏ primer hybridizes to the poly(A) tail of mRNA. It is‏ phosphorylated on the 5´ end to facilitate cloning of‏ cDNA) as primers for the synthesis of the first-strand‏ (Parstous, IRAN) as per manufacturer’s protocols.‏

Briefly, cDNA synthesize was performed in the presence‏ of random hexamer or oligo dT, then incubated at‏ 65ºC for 5 min, and followed by addition RT pre-mix,‏ incubation at 25ºC for 10 min, 50ºC for 60 min and 70ºC‏ for 10 min. PCR reaction was performed in a final volume‏ of 20 μL containing: 10 μL Taq pre mix, 2 μL‏ cDNA, 2 μL primer (forward and reverse) and water to‏ a final volume of 20 μL. The sequences of primers used‏ in this study were as follows: GAPDH (Glyceral‏ dehyde 3-phosphate dehydrogenase) Forward 5´-AGATGGTGAAAGTCGGAGTCA-3´, Reverse 5´-ATCA‏ TTGATGGCCACCACTTG-3´ was used as the housekeeping‏ gene. The forward and reverse primer for Bax‏ were designed as 5´-TTTGCTTCAGGGTTTCATCCA-3´ and 5´-CTCCATGTTACTGTCCAGTTCGT-3´, forward‏ and reverse primer for sall4 were as follow 5´-‏ CCAAAGGCAACTTAAAGGTTCAC-3´ and 5´-CCGTG‏ AAGACCAATGAGATCTC-3´. Following to the amplification,‏ the PCR products were electrophoresed in a‏ 2% agarose gel and were evaluated by green viewer‏ staining (20).

### 3.11. Statistical Analysis

Our data were expressed as means±SD. Analysis of‏ variance and significant differences were obtained by‏ SPSS 16 software, one-way ANOVA analysis. Values‏ of the *P*<0.05 were considered as significant.

## 4. Results

### 4.1. Effect of Chrysin on CT26 Cell Proliferation

**Figure 1:**
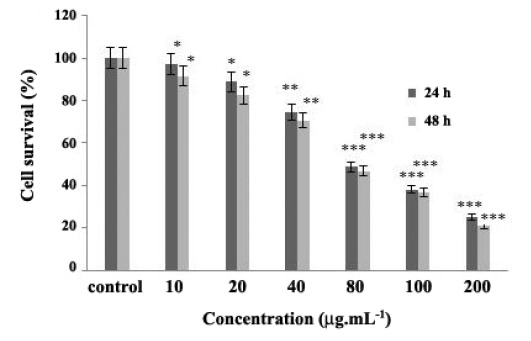
The inhibitory effect of chrysin on CT26 colon cancer cells viability. Cells were treated with a range of chrysin concentration (10, 20, 40, 80, 100, 200 μg.mL^-1^) for 24 and 48 h, then MTT assay was performed. Data represents as mean ± SD of three independent experiments. *p* value of *P<0.05, **P<0.01 and ***P<0.00 were considered significant

To determine the cytotoxic effect of chrysin, colon‏ cancer were treated with different concentrations of the‏ chrysin ranging from 10 to 200 μg.mL^-1^ for 24 and 48 h.‏ (Figure 1) shows that chrysin treatment inhibited CT26‏ cell proliferation as a dose dependent manner. The‏ growth rate of CT26 cells in response to 80 μg.mL^-1^ was‏ 48.75% and 46.68% at 24 h and 48 h, respectively. The‏ IC50 for chrysin was determined to be approximately 80‏ μg.mL^-1^ which reduced CT26 cells’ growth up to 50%‏ compared to the untreated cells.

### 4.2. Morphological Studies by Inverted and Fluorescence Microscope

**Figure 2:**
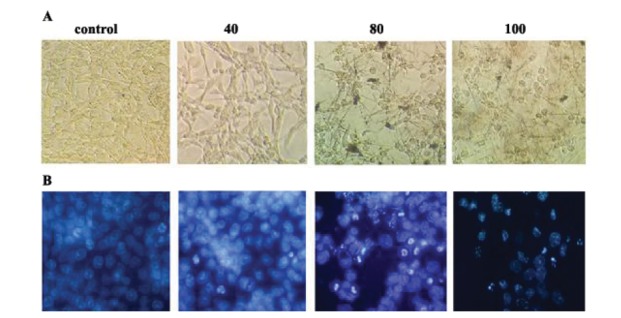
A: CT26 cells morphological changes following to the treatment of the cells with chrysin at different concentrations for 48 h. From the left: control cells, or cells without any treatment, and toward the right: cells that were treated with 40 μg.mL^-1^, 80 μg.mL^-1^, and 100 μg.mL^-1^ of chrysin for 48 h, respectively. B: The fluorescence profiles of the corresponding CT26 cells treated with the same concentration of chrysin and stained with DAPI. Arrows indicate DNA fragmentation in the corresponding treated cells group

DAPI staining is a reliable apoptotic assay in‏ chemoprevention studies and it helps to observe the‏ apoptotic changes at DNA level. As exhibited in‏ (Figure 2A), specific concentration of chrysin has‏ induced apparent apoptotic morphological alterations,‏ such as a reduction of cell volume, cell shrinkage, and‏ apoptotic body formation in CT26 colon cancer cells‏ showing that apoptosis has been triggered under exposure‏ with growth inhibitory concentration of the‏ chrysin. DAPI staining showed that chrysin has‏ induced chromatin condensation as a typical apoptotic‏ feature in the inhibitory concentrations of chrysin‏ (Figure 2B).

### 4.3. Detection of Apoptosis by PI Flow Cytometry

The apoptosis inducing effect of the chrysin was‏ analyzed using flow cytometry according to the exclusion‏ of PI by viable cells. As shown in the (Figure‏ 3A), chrysin treatment has significantly increased the‏ sub-G1 peak, which is an indicator of apoptosis induction‏ resulted from DNA fragmentation, and endonuclease‏ activation that is considered as a crucial anti-proliferative‏ mechanism of chrysin treatment.

### 4.4. AnnexinV FITC Assay

**Figure 3:**
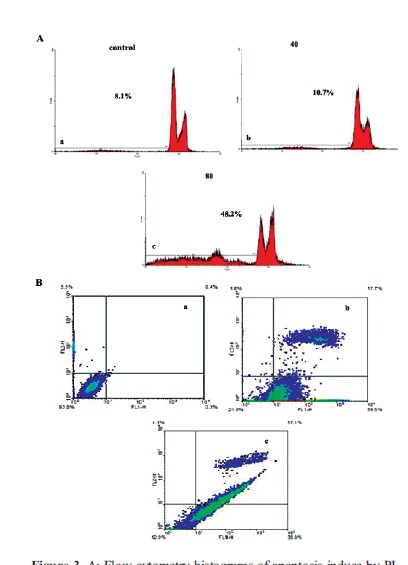
A: Flow cytometry histograms of apoptosis induce by PI method in CT26 cells, a: untreated cells, b: cells treated with 40 μg.mL^-1^ chrysin, and c: treated with 80 μg.mL^-1^ chrysin for 48 h. Apoptotic cell death can be quantified by DNA content frequency histograms (sub-G1 peak). B: Quantification of apoptosis cell death by Annexin-V/PI double-staining assay. Flow cytometry charts of CT26 cells, a: untreated or control, b: treated with 40 μg.mL^-1^ chrysin, and c: 80 μg.mL^-1^ chrysin, respectively

The apoptotic alterations of the plasma membrane can‏ be perceived through Annexin V-FITC (Fluorescein isothiocyanate)‏ staining (18). To verify the apoptotic morphological‏ changes as a result of treatment with chrysin (80‏ μg.mL^-1^), annexin V-FITC/PI staining was performed.‏ Flow cytometry diagrams determined that the percentage‏ of annexin positive cells were gradually increased as the‏ concentration of the chrysin was increased; from the live‏ cells, to an early apoptotic, the late apoptotic, as well as‏ necrotic cell death where it enhanced remarkably. Hence,‏ the percentage of apoptotic cells was 0.7% in the control‏ group, 77.2% in 80 μg.mL^-1^ and 85.7% in 100 μg.mL^-1^‏ treated groups, which is the hallmark of apoptosis induction‏ under treatment with IC50 concentration of the‏ chrysin on CT26 colon tumor cells (Figure 3B).

### 4.5. Determination of the Type of Apoptotic Pathway Induced by Caspase -3 and -9 Assay

**Figure 4:**
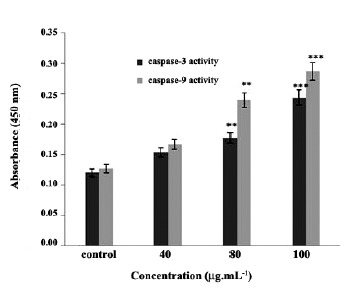
Caspase-3 and Caspase-9 activities in the CT26 cells treated with 40, 80, 100 μg.mL^-1^ of chrysin. Values are expressed as the mean ± SD of the triplicate experiment. Significant differences were compared with the control at *p < 0.05 by t-test

To determine the type of apoptotic pathway involved, the level of caspase -3 and -9 were calculated‏ colorimetrically. Our results elucidated that cytotoxic‏ effect of chrysin administrated through an increase in‏ the caspase-3 and caspase-9 activity in the treated cells‏ in a concentration-dependent manner as compared with‏ the control cells. Therefore, it could be concluded that‏ chrysin exerted its cytotoxicity effects on the CT26‏ colon cancer cells via intrinsic apoptotic pathway‏ (Figure 4).

### 4.6. Effect of Chrysin on the Tumor Volume

**Figure 5:**
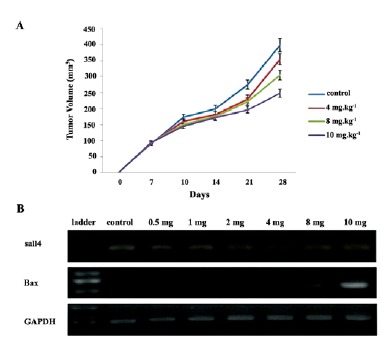
A: Relative variations in the tumors volume within different groups during 28 days. Tumor volumes were measured following to the exposure of mice to the 0.5, 1, 2, 4, 8, 10 mg.kg^-1^ chrysin and were monitored every day using a digital caliper, and relative variations of the growth inhibition of tumors in different groups were measured. B) Effects of chrysin on sall4 and Bax mRNA levels in CT26 cells. Transplanted BALB/c mice were treated with 0.5, 1, 2, 4, 8, and 10 mg.kg^-1^ chrysin which demonstrated the involvement of sall4 expression and pro apoptotic effect of chrysin on CT26 cells

As was shown, chrysin suppresses tumor cell‏ growth. We have further investigated its antitumor‏ effects in a mouse model of the colon cancer. As it is‏ shown in (Figure 5A), at the terminalexperiment, the‏ oral administration of chrysin at 8 and 10 mg.kg^-1^ has‏ led to a significant regression in the tumors’ volume as‏ compared to the control group treated with the vehicle‏ solution (i.e. PBS).

### 4.7. Effect of Chrysin on Sall4 and Bax mRNA Expression in vivo

To investigate the involvement of Bax in the induction‏ of apoptosis *in vivo* in the CT26 colon cancer cells,‏ cells were treated with 50% inhibitory concentrations of‏ chrysin, then expression of Bax family as proapoptotic‏ factors was assessed using RT-PCR. Figure 5B shows‏ that chrysin has up-regulated the level of Bax in a dose‏ dependent manner. As well, analysis of sall4 transcriptional‏ levels showed that treatment with chrysin has‏ down regulated sall4 mRNA level dose dependently in‏ colon cancer cells.

## 5. Discussion

In our study, the anticancer efficacy of the chrysin‏ against CT26 colon cancer cells was investigated. Our‏ observation revealed that chrysin possess moderate‏ cytotoxic effect on CT26 cells in a concentration‏ dependent manner. In addition, the findings from fluorescence‏ microscopy, Annexin-PI and caspase assays‏ showed that the cytotoxicity of chrysin was related to‏ the recruitment of the intrinsic pathway of apoptosis‏ cell death.

Natural products have been used as an alternative‏ medicine from the ancient time and plant-derived compounds‏ were considered as the safe modalities that‏ offer multitude benefits for preventing chronic diseases‏ such as cancer (21,22). Chrysin is a natural‏ flavonoid presented in many plant extracts, including‏ blue passion flower (*Passiflora caerulea*), honey, and‏ propolis (23). It is a natural, biologically active compound‏ that possesses potent anti-inflammatory, anticancer,‏ and antioxidant properties (24).

The pro apoptotic effect of chrysin has been reported‏ in breast carcinoma, cervical cancer, leukemia, lung‏ cancer (NSCLC), and colon cancer *in vitro* (9). A study‏ by Zhang *et al*. has demonstrated that chrysin and‏ tetraethyl bis-phosphoric ester (one of chrysin derivatives)‏ exhibited potential anti-cancer effects in human‏ cervical carcinoma cells (25).

The survey carried out by Shao *et al*. has revealed‏ that chrysin induced growth inhibition and apoptosis in‏ the cultured lung cancer; the A549 cells, along with activation‏ of AMP-activated protein kinase (AMPK) (26).‏ Recently, a synergism between doxorubicin and chrysin‏ administrated cancer cell death has been shown through‏ chemosensitizing these cells to chemotherapy via GSH‏ depletion in the cancer cells (27).

In this study, we have investigated the anti-cancer‏ effect of chrysin against colon cancer cells *in vivo*.‏ CT26 cell line was injected subcutaneously into mice‏ for allograft colon cancer model formation and assessment‏ of the anti-cancer efficacy of chrysin against colon‏ cancer. The tumor volume measurement has indicated‏ that chrysin (i.e., 4, 8, 10 mg.kg^-1^) dose dependently‏ reduces the tumor volume. In the present study we‏ attempted to further address the anti-cancer potential‏ of chrysin and the involved molecular mechanisms.‏ The *in vivo* results confirmed that regression of mouse‏ CT26 tumor volume under exposure to chrysin was‏ associated with apoptotic death via up regulation of‏ Bax mRNA level in concentration of 10 mg.kg^-1^.‏

The anti-cancer potential of chrysin has also been‏ reported in a variety of cell lines (28) and animals (29).‏ Other previous studies have shown that chrysin sensitizes‏ apoptosis induced cell death by tumor necrosis‏ factor (TNF), or tumor necrosis factor-related apoptosis-inducing ligand (TRAIL) in various human cancer‏ cells (30), suggesting the therapeutic potential of the‏ chrysin for treatment of human cancers. Izuta and his‏ colleagues have found that chrysin triggers caspase-3‏ activation, mitochondrial membrane depolarization‏ and mitochondrial cytochrome c release (31).

Pal-Bhadra and coworkers have shown the effect of‏ chrysin and its analogues on cell viability and cell‏ cycle analysis by MTT assay and flow cytometry.‏ They elucidated that chrysin possess potent *in vitro* anti-cancer activity via suppression of the cell proliferation,‏ induction G1 cell cycle arrest, along with the up‏ regulation of p21, in addition to reduction of cyclin D1‏ and Cdk2 protein levels (32).‏

Associated with the analysis of sall4 expression; in‏ most human malignancies, SALL4 might be over‏ expressed and potentially used as a diagnostic marker.‏ Similarly, Cao *et al* have reported the over expression‏ of SALL4 protein as a novel diagnostic marker for‏ germ cell tumors such as testis and ovary as well as‏ other metastatic germ cells’ tumors (33). Forghani‏ Fard *et al*. (2013) have reported that sall4 is one of the‏ genes involved in the colon carcinogenesis (34).

A well, in this study we assessed whether anti-cancer‏ effect of chrysin on CT26 cells is associated with‏ sall4 expression or not. Hence, RT-PCR analysis was‏ performed and the mRNA profile of sall4 has demonstrated‏ that chrysin exerts cytotoxicity on CT26 colon‏ tumor cells via down regulation of the sall4 expression.‏ ‏ However, sall4 down regulation could be considered‏ as a palliative marker for treatment of colon cancer.

In conclusion, we have elucidated that chrysin‏ inhibited the proliferation and induced apoptosis in‏ CT26 colon cancer cells and reduction of tumor volume‏ in animal model. Moreover, human clinical trials‏ are necessary to make it clear whether this flavonoid‏ could be proposed as a possible natural agent for the‏ prevention and treatment of the human colon cancer.

## Acknowledgments

This research was supported by Islamic Azad‏ University of Mashhad (Research Center of the‏ Applied Biology, Kharazmi Institute).
